# Fatigue-Induced HCP-to-FCC Phase Transformation Resulting in Two FCC-Zr Variants in Pure Zirconium

**DOI:** 10.3390/ma16186215

**Published:** 2023-09-14

**Authors:** Qing Jiang, Yao Chen, Qi Shuai, Fulin Liu, Lang Li, Chao He, Hong Zhang, Chong Wang, Yongjie Liu, Qingyuan Wang

**Affiliations:** 1 Failure Mechanics and Engineering Disaster Prevention Key Laboratory of Sichuan Province, College of Architecture and Environment, Sichuan University, Chengdu 610065, China; 2 Department of Mechanical Engineering, Kyushu University, Fukuoka 819-0395, Japan; 3 Institute for Advanced Study, Chengdu University, Chengdu 610106, China

**Keywords:** pure zirconium, high-cycle fatigue, fatigue-induced phase transformation, HCP-to-FCC phase transformation, variants

## Abstract

This study utilized transmission electron microscopy (TEM) and on-axis transmission Kikuchi diffraction (TKD) to investigate the fatigue-induced HCP-to-FCC phase transformation in industrial pure zirconium under a stress ratio of R = 0.1. The results show that fatigue damages result from phase deformations during cyclic loadings. The fatigue-induced FCC-Zr phases exhibit a B-type orientation relationship with the HCP-Zr matrix. Notedly, due to the different growth directions of Shockley partial dislocations relative to nucleation points, there are two FCC-Zr variants after the HCP-to-FCC phase transformation. The content of these two variants accounts for 65% and 35% of the total FCC-Zr, respectively, appearing as lamellae morphology embedded parallelly within the matrix. The distribution of the two variants includes isolated distribution and adjacent distribution. For the adjacent distribution, a twinning relationship is observed between the two variants. Meanwhile, as an intermediate transition stage of the HCP-to-FCC phase transformation, stacking faults are observed at the boundaries of the FCC-Zr lamellae. These findings offer insights into the microstructural features and formation mechanisms of fatigue-induced HCP-to-FCC phase transformation.

## 1. Introduction

Zirconium (Zr) and its alloys have found widespread applications in various fields such as chemical industry, automotive, aircraft engines, and biomedical materials [1,2,3,4] due to their excellent thermal conductivity, low thermal neutron absorption, outstanding corrosion resistance, and favorable mechanical properties, processability and weldability. In the chemical industry, zirconium alloys exhibit exceptional corrosion resistance to most of the commonly used chemical solutions, including organic and inorganic acids, strong alkalis, and certain molten salts. Consequently, they are widely employed in heat exchangers, reaction vessels, pumps, valves, and pipelines for transporting corrosive media, where they experience flow-induced stresses. In the field of biomedical applications, zirconium’s non-toxic and biocompatible nature makes it suitable for manufacturing surgical instruments and implants, as it exhibits good compatibility with muscles, bones, and other bodily tissues. During usage, these materials are subjected to prolonged fatigue loading, and due to the complexity of replacement procedures, they are required to have extended lifetimes. Therefore, understanding the fatigue behavior of zirconium and its alloys is a critical concern in their service processes.

In recent years, solid-state phase transformations in Zr materials have garnered increasing attention [5,6,7]. According to reports, Zr commonly appears in three different types of structures: a hexagonal close-packed structure (HCP, α-Zr), body-centered cubic structure (BCC, β-Zr), and simple hexagonal structure (ω-Zr) [3,8,9]. However, during the processes of thermal treatment [10] and plastic deformation [11,12], a unique phase with a face-centered cubic structure (FCC-Zr) has been observed in Zr and its alloys. Relevant simulation results show that the total energy of this FCC phase lies between α-Zr and β-Zr, confirming the existence of the FCC-Zr phase [10,12,13]. Similar FCC phases have also been found in other elements of the IVA group (Ti [14], Hf [15]). It is widely believed that the FCC phase is introduced through an HCP-to-FCC phase transformation. Beyond the dislocation slip and deformation twin, this phase transformation mechanism offers a third deformation mode to efficiently accommodate plastic deformation [16,17]. As reported [12,15,18,19], there are primarily two crystallographic orientation relationships (OR) between HCP-Zr and FCC-Zr: [1$\bar{2}$10]_HCP_//[110]_FCC_ and (0002)_HCP_//(1$\bar{1}$1)_FCC_ (i.e., B-type OR); and [0001]_HCP_//[001]_FCC_ and (10$\bar{1}$0)_HCP_//(110)_FCC_ (i.e., P-type OR). The B-type FCC transformation is believed to result from the gliding of Shockley partial dislocations (a/3[10$\bar{1}$0]) on alternate basal planes in the HCP matrix [10,12,14,15,20].

For a significant period, Zr has been regarded as a single-phase material with an HCP structure during various service periods. However, due to the stress fluctuations caused by the flow of fluids inside the pipes, the pipes have been subjected to a significant amount of cyclic loading. Obviously, Zr experiences plastic strain accumulation and fatigue damage. Our previous research shows that cyclic loadings induce the appearance of the FCC-Zr phase [21], indicating that the in-service Zr may not be a single-phase material but a potential dual-phase material. This could potentially have an impact on the fatigue performance of the material. As a newly emerging phase during the fatigue process, the relevant characteristics of the FCC-Zr phase remain unclear, highlighting the urgent need for further investigation.

In this study, we employed techniques including site-specific focused ion beam (FIB) milling, on-axis transmission Kikuchi diffraction (TKD), and transmission electron microscopy (TEM) to conduct comprehensive characterizations of fatigue-induced damage in pure Zr. We also provided a detailed discussion on the formation mechanisms of the variants with different orientations within the FCC-Zr phase. These findings are expected to provide valuable insights into the HCP-to-FCC phase transformation, carrying significant academic and safety implications.

## 2. Material and Methods

**Figure 1:**
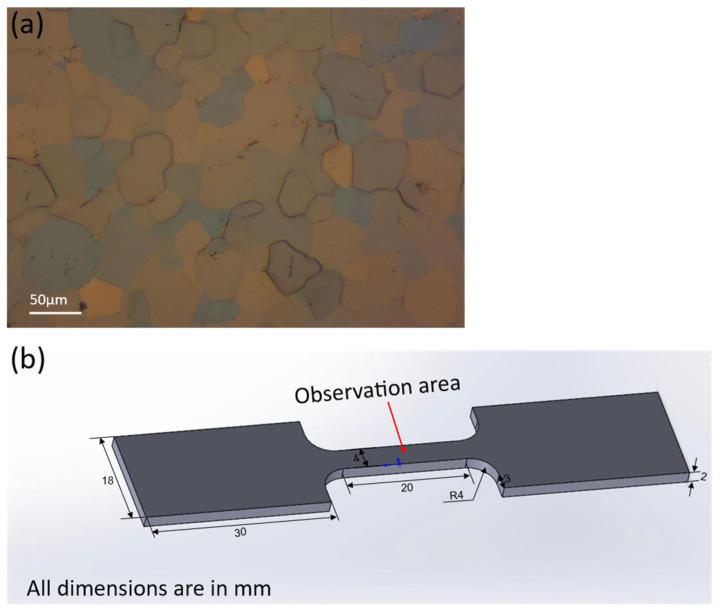
(**a**) Microstructure of industrial pure zirconium obtained using polarized light microscopy. (**b**) Dimensions of the fatigue test specimens and the observation area for fatigue damage.

Industrially pure zirconium with a nominal composition of Zr-0.99Hf-0.12O (mass fraction, %) was employed as the experimental material in this investigation. In order to minimize the impact of the surface roughness on fatigue behavior and achieve a smooth surface for comparing the microscopic structural changes in the specimen’s surface before and after fatigue testing, for each specimen before fatigue testing, grinding was performed on sandpapers of varying fineness to remove deeper scratches. Subsequently, mirror polishing was conducted using a polishing solution on a polishing machine, followed by chemical etching, and finally metallographic observation. Polarized light is an excellent contrast enhancement technique that outperforms traditional dark-field and bright-field illumination methods in terms of image presentation, particularly when applied to birefringent materials. Zr is a material with significant polarized light properties. This characteristic allows Zr to exhibit a pronounced contrast enhancement effect when imaged under polarized light microscopy, thereby providing a clearer display of the material’s microstructure. Through polarized light microscopy, it is possible to accurately differentiate and analyze the various orientations and forms of crystals, leading to a deeper understanding of Zr’s crystallographic characteristics and grain orientation distribution. This, in turn, offers a more precise perspective for the study of Zr material properties and performance. The polarized light microscopy in Figure 1a displays the grain microstructure, indicating the presence of equiaxed grains with sizes in the range of several tens of micrometers.

This study employed a high-frequency fatigue testing machine, model QBG-100, manufactured by the Changchun Qianbang Testing Equipment Co., Ltd. (Changchun, China) in 2012. This machine has been widely used for testing various metal materials’ resistance to fatigue fracture, determining the KIC values, and more. The fatigue testing was conducted following the international standard ISO 1099:2017 [22], under ambient temperature and atmospheric conditions. To minimize temperature variations during the fatigue tests and ensure a consistent room temperature environment, the testing facility was set up in an isolated indoor environment. The machine is equipped with a cold water circulation system, and a spiral cold air dryer system is also installed on-site. Figure 1b depicts the experimental dimensions based on both international standards and the machine design. During the fatigue loading process, the loading parameters were set according to the experimental standards, with a stress ratio of R = 0.1 and a constant stress amplitude. The fatigue tests were performed using sinusoidal cyclic loading at a frequency of 70 Hz. The specimens were subjected to 10^7^ cycles of loading, and if fatigue failure occurred during this period, the test was automatically terminated, and the fatigue life was recorded. After completing the fatigue tests, comprehensive examinations of the surface fatigue damage in the straight sections of the specimens were conducted using polarized optical microscopy (Olympus GX53, Olympus, Tokyo, Japan) and scanning electron microscopy (SEM, JEOL 6510, JEOL Ltd. Tokyo, Japan), as shown in Figure 1b. For specimens that experienced fatigue failure, side-surface observations were performed at a certain distance from the fracture surface to mitigate the influence of the main crack on fatigue damage. This observation area showed no significant plastic deformation. For specimens that did not fail even after 10^7^ cycles, a comprehensive observation of the whole surface was carried out. To ensure accurate observations, each specimen underwent alcohol ultrasonic cleaning and was dried with cold air.

After the surface observations, specific regions containing fatigue damage were selected on the side surfaces and cut using a wire saw. The samples were then secured onto the sample holder of a Helios G4 UX system, with the processed surface facing upward. The damaged locations were located using the secondary electron mode. To protect the sample’s surface from ion beam etching, a Pt coating was applied to the very top surface of the specimen. Subsequently, a dual-beam FIB/SEM system (ZEISS, Jena, Germany, CrossBeam Laser 540) was used to cut the region with Pt coating. Thin foils (approximately 100 nm thick) were extracted from the designated damaged areas. Once the cutting was completed, the samples were transferred to copper grids, welded, and subjected to thinning processes. After the sample preparation, the copper grids were placed in high-vacuum membrane boxes for storage. For collecting and analyzing the foils, on-axis TKD (BRUKER QUANTAX CrystAlign 400i, BRUKER, Billerica, MA, USA) and TEM (FEI Talos F200X, FEI, Eindhoven, The Netherlands, accelerating voltage: 200 kV) were utilized. The collected TEM images were analyzed using the DigitalMicrograph (Version 3.22.1461.0) software to obtain the results, such as fast Fourier transformation (FFT) and inverse fast Fourier transformation (IFFT). The collected TKD data were analyzed using AztecCrystal software (Version 2.1) to aid in understanding the mechanisms of localized fatigue damage.

## 3. Results and Discussion

### 3.1. Parallel Lamellae within Fatigue Damage Area

**Figure 2:**
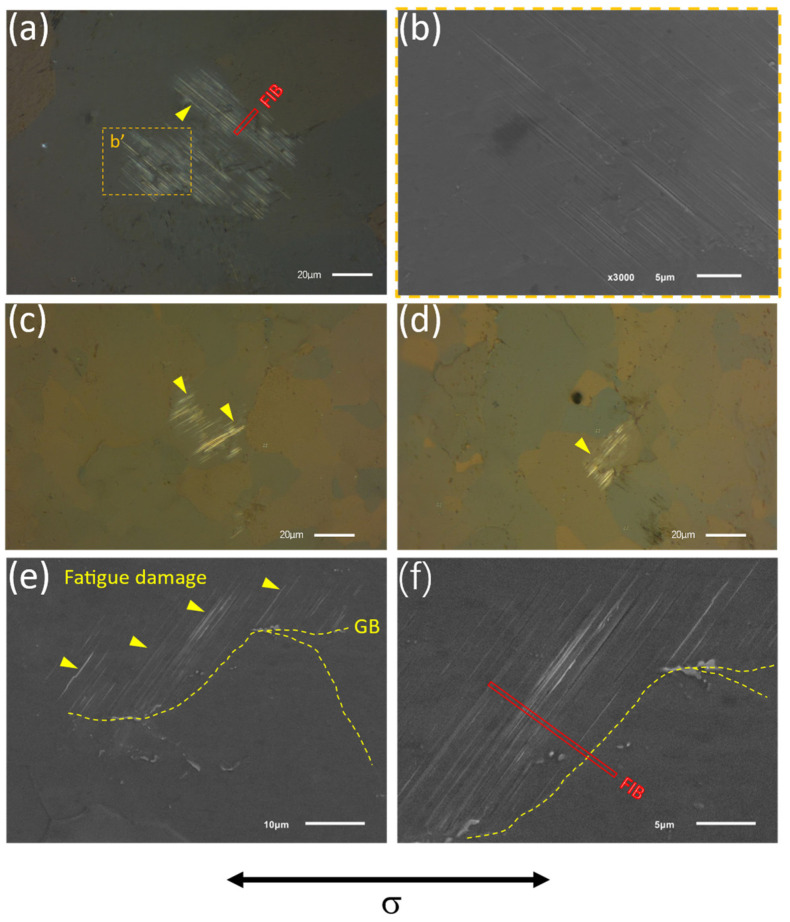
Parallel bands of fatigue damage were seen on the specimen’s surface, as indicated by the yellow arrows: (**a**,**b**) Observations of failed specimens (σ_a_ = 130 MPa, N_f_ = 1.6919 × 10^6^ cycles). Where (**b**) is an enlarged image corresponding to b′ in (**a**). (**c**–**f**) Observations of surviving specimens (σ_a_ = 120 MPa, N_f_ = 1.0 × 10^7^ cycles).

Figure 2a shows the fatigue damage on the surface of the tested specimen that failed after 1.6919 × 10^6^ cycles under a cyclic stress amplitude of 130 MPa. Figure 2b shows the enlarged view of the fatigue damage in Figure 2a. The fatigue damages are parallel to each other, with varying lengths, and terminated at the grain boundaries. For the specimens subjected to 1 × 10^7^ cycles at a cyclic stress amplitude of 120 MPa without failure, they exhibit similar fatigue damage characteristics, as shown in Figure 2c–f. Figure 2c–f display the optical and SEM images of the fatigue damage, respectively. The images indicate that these damages occur within individual grains and exhibit distinct differences from the surrounding grains. Generally, local fatigue damage accumulation occurs at low stress amplitudes under cyclic loading. This damage accumulation leads to the nucleation and initiation of fatigue cracks [23,24,25]. Then, fatigue cracks propagate and, ultimately, result in a fracture. Therefore, investigating the fatigue damage mechanism is crucial for prolonging the fatigue life and ensuring the safety assessment. The following sections will provide a detailed characterization and discussion of the fatigue damages.

**Figure 3:**
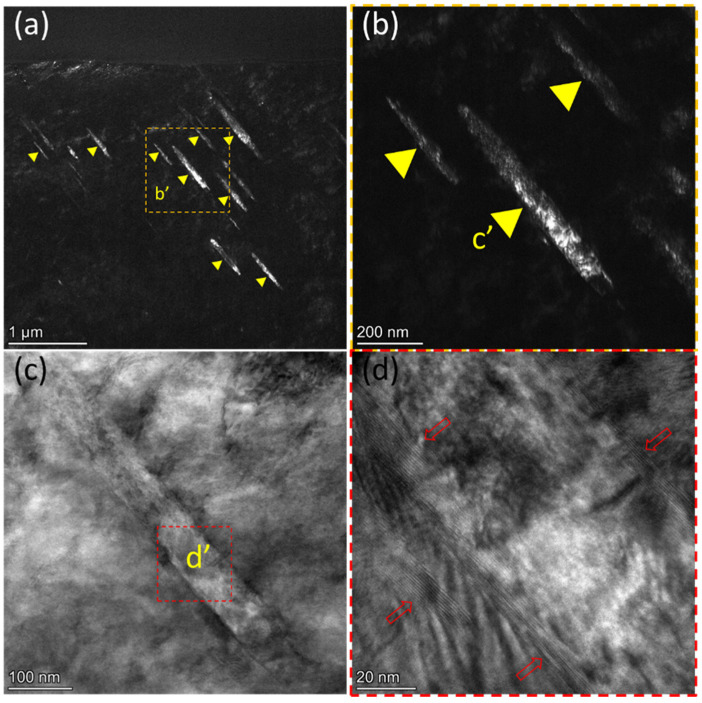
Parallel lamellae within fatigue damage area: (**a**) Dark-field TEM image of the thin foil extracted from Figure 2f shows numerous parallel lamellae indicated by yellow triangular arrows. (**b**) The magnified dark-field image from b′ in (**a**) reveals different diffraction contrasts within and between the lamellae. (**c**) Corresponding magnified bright-field image of the c′ lamellae in (**b**). (**d**) High-resolution image of the d′ region in (**c**), showing parallel fringes along the long-axis interface on both sides of the lamellae, as shown by the red arrow.

To investigate the relevant characteristics of fatigue damage, we employed FIB technology to extract thin TEM foils from the damaged region shown in Figure 2f. Figure 3a,b present typical dark-field TEM images beneath the sample surface. Within the HCP-Zr matrix, numerous lamellae are observed and indicated by yellow arrows. Their widths are about tens of nanometers, while their lengths range from tens to hundreds of nanometers. Obviously, these lamellae exhibited a high aspect ratio, and their thickness increased with the length. Despite the varying spacing between the lamellae, their long axes were mutually parallel. Additionally, they exhibited a distinct contrast with the HCP-Zr matrix.

Figure 3b displays an enlarged view of the lamellae shown in Figure 3a. In the dark-field TEM image, non-uniform contrast is observed within the lamellae, along with variations in the contrast intensity between adjacent lamellae. Figure 3c,d display the bright-field and high-resolution TEM images, respectively, corresponding to the lamellae shown in Figure 3b. In Figure 3c, straight interfaces are shown between the lamellae and the surrounding matrix. With the further examination in the high-resolution TEM image (Figure 3d), it revealed the presence of lattice fringes along the interfaces, as indicated by the red arrows. Therefore, within the fatigue damage region, the mutually parallel lamellae are expected to have crystallographic characteristics in relation to the matrix.

### 3.2. Deformation Twinning in Parallel Lamellae

**Figure 4:**
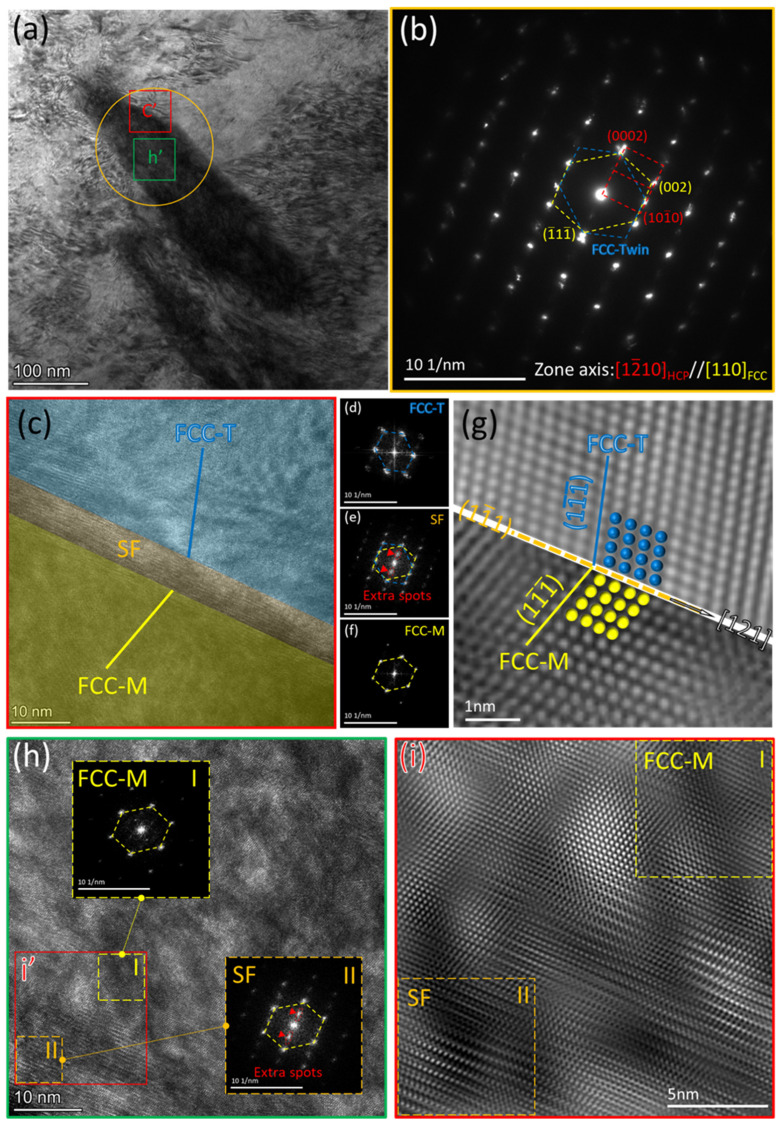
Characterization of deformation twins in the FCC-Zr using TEM: (**a**) Bright-field TEM imaging of the thin foil, where c’ and h’ respectively represent the ranges of the high-resolution images (**c**) and (**h**). (**b**) SAED pattern obtained from the region containing the lamellae and the matrix (highlighted by the orange circle in (**a**)), showing FCC twin-oriented phases and the matrix HCP phase. (**c**) High-resolution TEM (HRTEM) image taken from the rectangular region c′ in (**a**). (**d**–**f**) Corresponding FFT patterns of the respective areas in (**c**). (**g**) High-resolution corresponding image of the FCC deformation twins. (**h**) High-resolution image taken from the rectangular region h′ in (**a**). (**i**) Enlarged image of the i′ region within (**h**), where I and II respectively represent the areas inside the lamella and at the boundary.

In order to clarify the microstructural features of the parallel lamellae, a specific region was examined in detail, as shown in Figure 4. Figure 4a presents the bright-field TEM image of the selected region. Figure 4b shows the selected area electron diffraction (SAED) pattern, resulting from the circled region shown in Figure 4a. The selected area contains both the lamellae and the matrix. The SAED result revealed three sets of diffraction spots: one corresponding to the matrix with the HCP-Zr structure and the other two sets representing the mutually twinned FCC structures. After calibration, the twin boundary was determined to lie along the (1$\bar{1}$1) plane of the twinned FCC structures (Figure 4b,g). Based on this SAED pattern, the OR between the HCP-Zr matrix and the FCC twin layers was identified as [1$\bar{2}$10]_HCP_//[110]_FCC_ and (0002)_HCP_//(1$\bar{1}$1)_FCC_. This orientation is congruent with the orientation of the basal type (B-type) [12,15,18,19], indicating that the lamellae should be the FCC-Zr phase. This type of HCP-to-FCC phase transformation has been reported in cold-rolled Hf [15], compressed Ti [14,18], and cold-rolled Zr [11,12]. As is well known, the traditional plastic deformation mechanisms in HCP crystals include dislocation slip and twinning. Due to the relatively low symmetry of HCP-Zr crystal structures, there are fewer than five independent slip systems, most of which are confined to the <a> direction [26]. To accommodate plastic deformation, especially the strain along the crystallographic c-axis, HCP twinning is formed in addition to dislocation slip. This twinning helps facilitate uniform plastic deformation in the material. However, the activation of HCP twinning is influenced by a combination of specific stresses and crystal orientations, and it is difficult to form HCP twins in small grain sizes [27,28]. Therefore, when twinning is limited, the HCP→FCC phase transformation takes place. At the atomic level, both slip and twinning involve atomic displacements along specific vectors. In the case of the B-type HCP→FCC phase transformation, Shockley partial dislocations with a Burgers vector of a/3<10$\bar{1}$0> slip on every other (0001) basal plane, causing the gradual transition of the HCP structure to the FCC structure [10,12,14,15,20]. This transition is achieved through atomic displacements. Additionally, this phase transformation results in strain along the c-axis, similar to the effect of twinning [11]. Therefore, besides slip and twinning, the HCP-to-FCC phase transformation can also be considered as an additional deformation mode in HCP crystal structures.

Past research has demonstrated that methods to obtain FCC phases in bulk materials with an HCP structure can be classified into two main categories. One involves the precipitation of the FCC phase within the material matrix during the solidification or heat treatment of metals or alloys. The other method involves inducing the HCP-to-FCC phase transformation through plastic deformation of metals or alloys, triggered by stress. In other words, the HCP-to-FCC phase transformation can be achieved through both heat treatment [10] and stress-induced mechanisms [19]. According to current reports, stress-induced FCC phase transformation is the primary method for obtaining the FCC phase. In this study, mirror polishing and metallographic observation were performed on each fatigue specimen before loading, and no corresponding plastic deformation or damage was observed. Subsequent observations of fatigue-induced damage indicated that surface plastic deformation and damage occur during the fatigue loading process. This outcome also underscores that the HCP-to-FCC phase transformation discussed in this paper is solely driven by mechanical stress and not influenced by heat treatment. Regarding stress-induced HCP-to-FCC phase transformation, shear [29], tension [20], and compression stress [18] have been identified as contributing factors. Zhang et al. [19] also provided direct evidence of stress-induced HCP-to-FCC phase transformation using the Vickers microhardness indentation, thereby supporting the findings. Furthermore, the research has shown that plastic strain or the strain rate significantly influences the phase transformation [20], and grains positioned near the free surface are more susceptible to experiencing phase transformation [30]. In this study, a positive stress ratio is applied with cyclic tensile–tensile loading, similar to a tension stress state and loading method. This configuration, characterized by a higher strain rate and the accumulation of long-duration localized plastic deformation, readily induces the HCP-to-FCC phase transformation in grains located at free surfaces.

Figure 4c shows the high-resolution TEM images at the lamellae interface, revealing the twin relationships. On both sides of the interface, the FCC-M area in Figure 4c corresponds to the set of diffraction spots with the yellow link in Figure 4b, while the FCC-T area in Figure 4c corresponds to the set of diffraction spots with the blue link in Figure 4b. Figure 4d–f give the corresponding FFT patterns of the region in Figure 4c. Figure 4g provides the local high-resolution twin relationship, further revealing the twinning orientation relationship between the two regions with the FCC structure. For the twin structure, the twin plane is determined to be the (1$\bar{1}$1) plane, which is the dense-packed plane in the FCC structure. The twinning direction is along the [121] direction. It is typical {111} <112> twinning in FCC materials [31]. This deformation twinning has been reported in the HCP-to-FCC phase transformations [16,32,33]. It is shown that the presence of twinning could reduce the critical energy, required for the phase transformation [34]. As a result, this work suggests that cyclic loading can produce FCC deformation twins.

In addition, near the twin boundary shown in Figure 4c, fringes similar to those in Figure 3d are observed. To determine the structure of these fringes, they were analyzed by fast Fourier transform, as shown in Figure 4e. In Figure 4e, some regular additional spots are observed, as indicated by the red arrows. These additional spots are interspersed among the normal diffraction spots of the FCC structure. The HCP-to-FCC phase transformation is typically accomplished through the motion of Shockley partial dislocations. This process is accompanied by the nucleation of intermediate structures [35,36,37], such as the R-phase, H-phase, and several periodic structures [36,37,38]. These intermediate structures have been found to facilitate the phase transformation, particularly for the FCC twinning [16,32,33]. Figure 4h,i show the high-resolution TEM images surrounding the lamellae boundary. It is found that numerous stacking faults are present near the lamellae boundary. Stacking faults can result from the interaction of partial dislocations on adjacent crystallographic planes. Partial dislocations are a type of dislocation that involves a limited distortion of the crystal lattice. They can be thought of as dislocations with a smaller Burgers vector (the magnitude and direction of lattice distortion). The accumulation of these partial dislocations can lead to a deviation from the perfect stacking sequence, resulting in a stacking fault. On the other hand, as shown in Figure 4i, I and II represent the regions within the lamella and at the boundary, respectively. The corresponding Fourier transform reveals that in the interior area I of the lamella, a typical FCC structure is evident without any stacking faults. In the boundary region II of the lamella, diffraction spots consistent with those in Figure 4e are present. It is suggested that the stacking faults correspond to the additional diffraction spots in Figure 4e,i, marked by the red arrows. It is worth noting that stacking faults are local defects that affect a small portion of the lattice and have a limited scope. Phase transformation refers to the change in a material from one crystal structure to another. It involves the rearrangement of atoms within the lattice to form a new structure with different symmetry. However, stacking faults can serve as nucleation sites for phase transformation. Changes in the atomic arrangement around stacking faults can create conditions favorable for the initiation of phase transformation. Stacking faults introduce interfaces between regions with different stacking sequences or atomic arrangements. These interfaces can serve as nucleation sites for the formation of new phases during the phase transformation process. Additionally, stacking faults can alter the local thermodynamic conditions in their vicinity. This may affect the driving force for phase transformation, leading to the occurrence or suppression of certain transformations to varying degrees. Therefore, these periodic spots represent an intermediate transitional structure during the phase transformation, which corresponds to stacking faults [39].

TEM characterizations show that the parallel lamellae are the FCC-Zr phase within the HCP-Zr matrix. Meanwhile, deformation twinning occurs as a result of the HCP-to-FCC phase transformation. Additionally, stacking faults near the lamellae boundary are considered as an intermediate structure during the HCP-to-FCC phase transformation. However, it should be noted that TEM characterization is limited to a local analysis, due to the limited target area. Regarding the FCC-Zr lamellae, their content, distribution, and possible variants remain unclear. Therefore, it is essential to conduct characterization on a larger scale, resulting in statistically significant characterization.

### 3.3. Two FCC-Zr Variants of the B-Type HCP-to-FCC Phase Transformation

Deformation twins are reportedly seen during the phase transformation, in accordance with pertinent studies [16,32,33], which is congruent with the results of this study. The traditional electron backscatter diffraction (EBSD) approach is not adequate for analyzing the FCC-Zr phase in this work due to its nanoscale size. This is because it is difficult to characterize the nanoscale FCC-Zr phases using the conventional EBSD approach, which has a spatial resolution limit of approximately 50–100 nm [40], making it challenging to characterize the nanoscale FCC-Zr phases. As a result, most current studies primarily focus on TEM characterization for the phase transformation. Therefore, there have been few studies on the large-area characterization of the phase transformation.

A spatial resolution better than 10 nm can be attained using the TKD technique, according to reports [41]. The standard TKD configuration has been enhanced by the on-axis TKD technique described by Fandenberger et al. in 2016 [42]. This advanced method produces an increased scattering electron intensity and resolution because the scintillator detector is positioned below the target foil sample and perpendicular to the electron beam. This enables highly effective characterization of the nanoscale lamellae of the FCC-Zr phases in this study.

**Figure 5:**
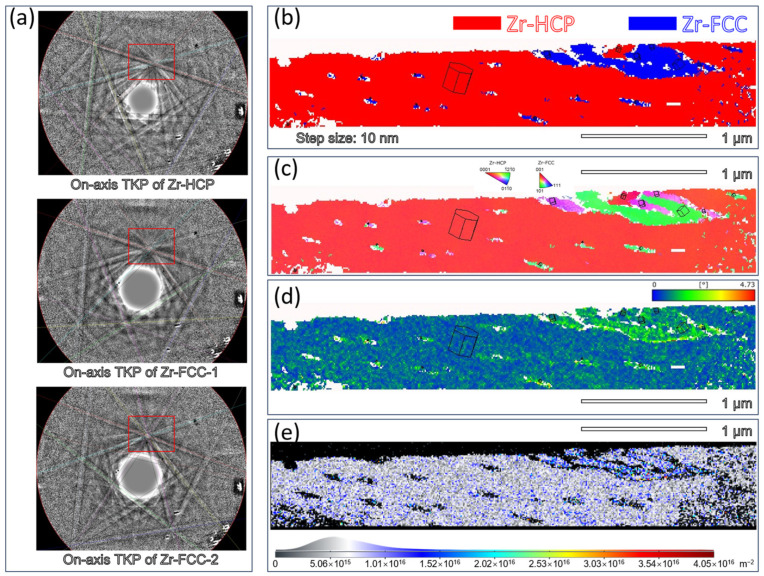
Identification and characterization of two orientations of FCC lamellae extracted from Figure 2a through on-axis TKD: (**a**) Corresponding Kikuchi diffraction patterns. (**b**) Phase distribution images show the presence of both the HCP matrix and the transformed FCC phase. (**c**) Inverse pole figure (IPF||Y0), showing the presence of two orientations of FCC phases. (**d**) Corresponding to the KAM distribution in (**b**), showing the local micro-strain around the transformed FCC phase with two orientations. (**e**) The distribution of GNDs reveals the accumulation of dislocations around the interface between the HCP matrix and the transformed FCC phase, indicating a higher dislocation density within the FCC phase.

Figure 5 shows the on-axis TKD results with a step size of 10 nm. The thin foil sample is extracted from the position shown in Figure 2a. In Figure 5c, the inverse pole figure (IPF) shows the presence of two variants of the FCC-Zr phases. The corresponding Kikuchi diffraction patterns are displayed in Figure 5a. The bright spots in the pattern’s center indicate the collection of the Kikuchi pattern information at the strongest signal position. This is a characteristic of the on-axis TKD technique. The parallel alignment of the patterns along the same line indicates the absence of distortion in the collected Kikuchi patterns. Compared to the off-axis TKD technique, the advantages of on-axis TKD include a faster collection speed, enhanced accuracy, and a higher calibration rate [43]. In this research, according to the on-axis TKD results, the sufficient diffraction patterns also confirm that the parallel lamellae are the FCC-Zr phases. Furthermore, it should be noted that there are two oriented FCC-Zr structures. According to the Kikuchi patterns shown in Figure 5a, the two FCC-Zr Kikuchi patterns show special orientation relationships with the HCP-Zr matrix, as highlighted by the red box.

In Figure 5b, the red region represents the matrix of the HCP-Zr phase, while the blue region represents the FCC-Zr phase. It can be observed that the larger surface area of the FCC-Zr phase in Figure 5b exhibits different colors in the inverse pole figure (IPF) map shown in Figure 5c. It means that the FCC-Zr phase exhibits two distinct orientation relationships. There are two variants of the FCC-Zr lamellae. On the other hand, combining the phase image, the content of the FCC-Zr phase is estimated to be approximately 9%. The content of the two variants accounts for 65% and 35% of the total FCC-Zr, respectively. Thus, the on-axis TKD technique is reliable for the identification of the nanoscale FCC-Zr phase and also the orientation relationships. Furthermore, the on-axis TKD technique offers a broader characterization area, when compared to TEM characterization. The results in this study not only strongly support the TEM characterization findings but also possess statistical significance.

The Kernel Average Misorientation (KAM) distribution for the area depicted in Figure 5b is presented in Figure 5c. Based on the variations in the local lattice orientation, the KAM distribution represents the localized plastic deformation. Higher KAM values in a region suggest a greater degree of regional strain. The findings reveal a localized misalignment at the boundary between the HCP-Zr matrix and the FCC-Zr phase, which points to a local strain gradient. This is a feature of the stress-induced phase transition from the HCP to the FCC [19]. The distribution of Geometrically Necessary Dislocations (GNDs) in Figure 5e, on the other hand, resembles the KAM distribution and suggests a larger dislocation density in the FCC-Zr phase. It is well-known that the HCP-Zr has only four independent slip systems, and at room temperature, slip deformation in the HCP phase primarily occurs along the prismatic slip systems [26]. According to the von Mises criterion, a minimum of five independent slip systems is required for plastic deformation in metal materials. Therefore, the plastic deformation capability of HCP zirconium is limited [44]. In contrast, the FCC structure theoretically provides 12 independent slip systems [45,46]. These slip systems are relatively easier to activate, resulting in a higher dislocation density. Additionally, the formation of the FCC phase within the matrix introduces FCC/HCP interfaces, which is theoretically equivalent to grain refinement. As a result, the strength and plasticity of the material are expected to be enhanced to some extent after the phase transformation [20,47]. Simultaneously, there is a specific lattice expansion during the phase transformation. This enables the phase transformation to effectively accommodate strain along the c-axis direction of the HCP phase, similar to the effect of tensile twinning [11]. This also results in a change in material volume, analogous to deformation twinning. The FCC phase can be considered a form of volume defect [48]. Numerous previous studies have indicated that large stress concentrations exist around deformation twin boundaries. During cyclic deformation, these twin boundaries eventually evolve into preferred locations for fatigue crack initiation in HCP materials [49,50]. In summary, in order to effectively accommodate plastic deformation, a phase transformation occurs during the loading process, leading to a certain degree of improvement in the material’s plasticity and strength. However, because of the accumulation of plastic deformation, stress concentration and strain mismatch can occur at corresponding interfaces, inducing the initiation of fatigue cracks and thereby affecting the material’s fatigue performance [21].

From Figure 5b,c, it is evident that there are some zero-solution regions surrounding the FCC-Zr boundaries. This is attributed to the fact that although the on-axis TKD technique allows high-resolution characterization with certain advantages, it still requires samples with high quality. This includes ensuring sufficient thinness to allow electron beam penetration and the absence of amorphous regions. Imperfections in the thin foil sample will present challenges in obtaining perfect signals. Furthermore, the TEM results in Figure 4 also reveal the presence of an intermediate transitional structure near the lamellae boundaries. There is a significant presence of stacking faults. These stacking faults, along with the dislocations within the FCC phase [20], may also contribute to the imperfect signal surrounding the FCC-Zr boundaries.

**Figure 6:**
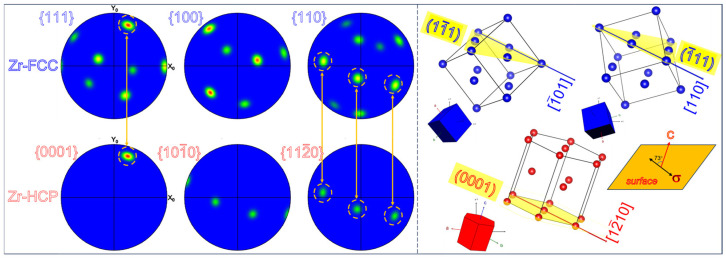
The total orientation relationship between the HCP matrix and the FCC phase is shown by pole figures and a schematic crystal lattice, suggesting a B-type phase transition.

The crystal lattice picture and pole in Figure 6 further identify the phase transformation. The converted FCC phase and the HCP matrix have the following orientation relationship: The arrows point to <1$\bar{2}$10>_HCP_//<110>_FCC_ and {0002}_HCP_//{1$\bar{1}$1}_FCC_. This orientation relationship is referred to as the B-type orientation relationship, which is consistent with the TEM results shown in Figure 4. According to the crystal lattice figure and the loading direction, it can be calculated that the angle between the crystal cell’s c-axis and the stress direction is approximately 73°. Calculations indicate that the activation of specific predominant deformation mechanisms in zirconium’s plastic deformation process primarily depends on the angle between the crystal cell’s c-axis and the tensile axis [51,52]. When this angle is approximately 73°, it is less conducive to the generation of HCP tensile twinning [51]. Additionally, there is a competitive relationship between stress-induced HCP-to-FCC phase transformation and HCP twinning [53]. When twinning is suppressed, phase transformation is promoted [15]. Therefore, under specific grain orientations and loading conditions, phase transformation is favored.

For Zr, it is at 863 °C that the β-Zr phase with a BCC structure transforms into the α-Zr phase with an HCP structure. During the phase transformation from β-Zr to α-Zr, the β-Zr phase and α-Zr phase follow the Burgers OR, i.e., {110}_β_||{0001}_α_ and <111>_β_||<$\bar{2}$110 >_α_ [46]. The dense-packed planes of the α-Zr phase align parallel to the dense-packed planes of the β-Zr phase, and the dense-packed directions are also parallel. Based on crystallographic theory, the theoretical β-to-α phase transformation can result in 12 different α variants. Similar to the BCC-to-HCP phase transformation at an 863 °C high temperature, the HCP-to-FCC phase transformation also presents 12 variants theoretically. This is due to the inherent symmetry between the parent and daughter phases. For the P-type OR, multiple variants have indeed been observed in several experimental studies [54,55,56], providing evidence for the correctness of theoretical variants. However, for the B-type OR, the current research is predominantly focused on TEM characterizations, in which the observed variants mainly consist of adjacent deformation twins. It should be noted that the variants resulting from the B-type phase transformation have rarely been reported.

Based on the results from TEM and on-axis TKD characterizations, it has been found that there are two orientation relationships between the FCC-Zr lamellae and the HCP-Zr matrix (Figure 5b,c). This indicates that two FCC-Zr variants are generated during the HCP-to-FCC phase transformation process. A subsequent investigation will focus on these two variants.

**Figure 7:**
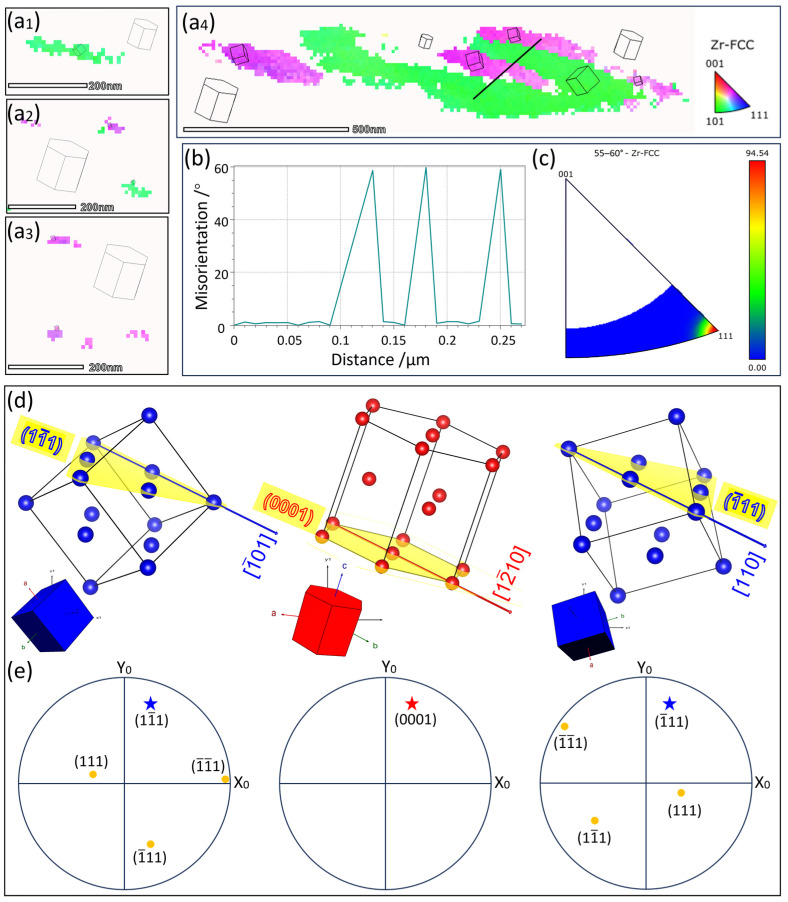
Analysis of the two variants of the B-type phase transformation through on-axis TKD characterization: (**a_1_**–**a_4_**) Inverse pole figure of the FCC-Zr (IPF||Y0), displaying the distribution characteristics of the two FCC variants. (**b**) Misorientation of the line in (**a_4_**). (**c**) Rotation angles and rotation axes of adjacent regions in (**a_4_**). (**d**,**e**) The orientation relationships between the two converted FCC phases and the HCP matrix are shown using a schematic lattice and the associated pole representations.

According to the on-axis TKD results, Figure 7(a_1_–a_4_) shows the inverse pole figure (IPF||Y0) maps of the two obtained FCC-Zr variants. The two FCC-Zr variants are distributed either separately or adjacently, and the variant with an adjacent distribution shows larger sizes. Additionally, in Figure 7(a_1_–a_4_), it can be observed that the long axes of the FCC-Zr are mutually parallel. This observation is consistent with the TEM findings. Relevant research has indicated that the long axes of the B-type FCC-Zr lamellae are parallel to the basal plane of the HCP matrix [11,12,15]. As a result, the long axes of the B-type FCC-Zr lamellae within the same HCP grain are mutually parallel. The characterization results presented in this paper are in alignment with this established conclusion. Figure 7b presents misorientation angle maps along the lines in Figure 7(a_4_) and the distribution of the rotation axes within the measured area. The results show that the misorientation angles along the lines are approximately 60°. The IPF in Figure 7c indicates a rotation angle range of 55° to 60° within the measured area, predominantly featuring <111> rotation axes. This suggests the presence of numerous 60° <111> twin boundaries within the measured area, as shown in Figure 7(a_4_). Notably, due to the influence of dislocations, the actual rotation angles may not be exactly 60° but slightly less, resulting in a certain degree of deviation at the interfaces [15]. Figure 7d,e illustrate the spatial orientation maps of the two variants and the matrix, along with their corresponding pole figures. The orientation relationships are (0001)_HCP_//(1$\bar{1}$1)_FCC1_//($\bar{1}$11)_FCC2_ and [1$\bar{2}$10]_HCP_//[$\bar{1}$01]_FCC1_//[110]_FCC2_. These orientation relationships are consistent with the B-type phase transformation. In conclusion, the on-axis TKD characterization results, in a statistical context, indicate the existence of two FCC-Zr variants during the B-type phase transformation.

### 3.4. Formation Mechanism of the Two FCC-Zr Variants

As mentioned earlier, both TEM and the on-axis TKD characterizations have revealed two FCC-Zr variants after the B-type phase transformation. Specifically, these two FCC-Zr variants can be distributed separately or adjacent to each other, forming deformation twins, with the adjacent distribution of the FCC-Zr variants exhibiting a larger size. Regarding the B-type phase transformation process, the gliding of the Shockley partial dislocations (a/3[10$\bar{1}$0]) on every other basal plane in the HCP-Zr matrix has been recognized as the transformation mechanism [10,12,14,15,20]. The phase transformation mechanisms for the two FCC-Zr variants will be discussed accordingly.

**Figure 8:**
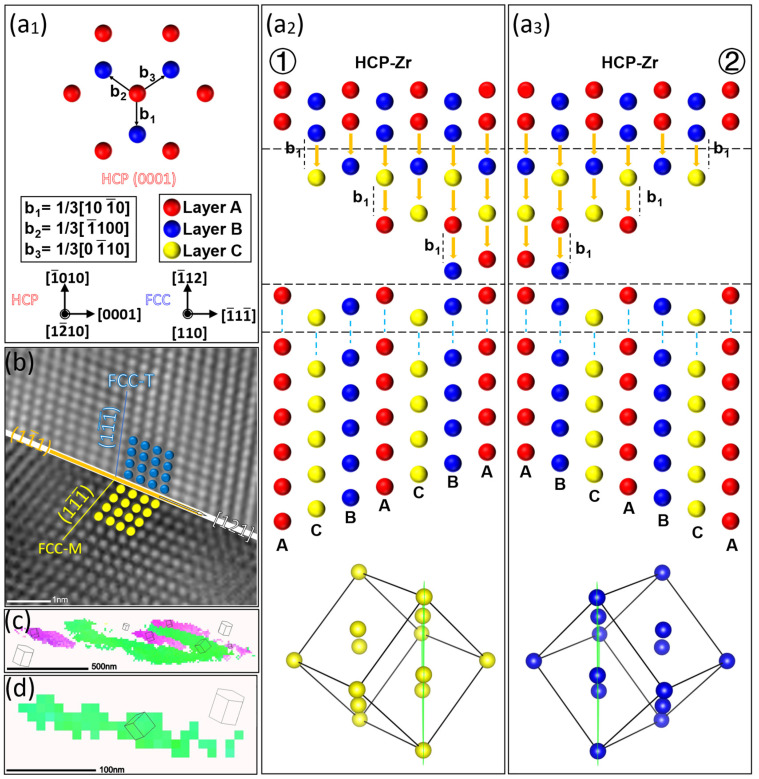
Formation Mechanism of FCC Phases with Different Orientations. (**a_1_**) The schematic diagram of partial dislocations in HCP-Zr. (**a_2_**,**a_3_**) Schematic diagram of the phase transformation process at different nucleation sites, where numbers 1 and 2 represent two different nucleation sites, and A, B, and C represent different layers. (**b**,**c**) Deformation twins in the FCC-Zr. (**d**) Individually distributed FCC lamellae.

Figure 8(a_1_) illustrates the schematic diagram of the partial dislocations in the HCP-Zr matrix. As indicated in Figure 8(a_2_), the HCP-Zr’s initial stacking order is ABABAB. The numbers 1 and 2 represent two distinct nucleation sites. During the cyclic loadings, the partial dislocations nucleate at points 1 and 2, respectively. Subsequently, they propagate by sequentially activating and gliding along the (0001) planes on one side of the nucleation point, every other layer, in the same direction. In particular, when the Shockley partial dislocations of nucleation point 1 open on every other layer of the basal plane to the right of the nucleation point, the FCC structure with the stacking sequence ACBACB is finally formed. Conversely, when the Shockley partial dislocations of nucleation point 2 open on every other layer of the basal plane to the left of the nucleation point, that is, in the opposite direction, the FCC structure with the stacking sequence ABCABC is finally formed. Furthermore, during the subsequent growth process, if the two nucleation points are sufficiently close, they will eventually make contact at the intermediate position. This leads to the formation of deformation twins, as shown in Figure 8b,c. On the other hand, if the two nucleation points are farther apart and result in a smaller-sized FCC-Zr, the two variants will form separate distributions, as depicted in Figure 8d. In summary, the different orientations and deformation twins of the FCC-Zr are related to the direction in which the Shockley partial dislocations activate on either side of the nucleation points.

**Figure 9:**
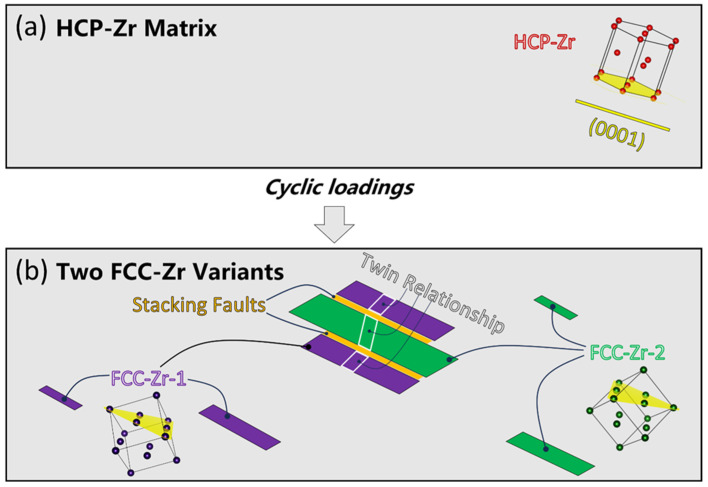
Schematic illustration of the HCP-to-FCC phase transformation after cyclic loadings. (**a**) Original microstructure of the HCP-Zr matrix before cyclic loadings. (**b**) Fatigue-induced phase transformation resulting in the two FCC-Zr variants.

In order to provide a better depiction of the two variants within the phase transformation and to comprehend their formation mechanisms, a simplified schematic diagram is presented in Figure 9, based on the TEM observations and on-axis TKD analysis as described earlier. During the process of fatigue loading, the cyclic loads activate Shockley partial dislocations at different locations, as illustrated in Figure 9a. These dislocations nucleate and continuously glide, leading to the growth of the FCC phase. In other words, fatigue-induced stresses drive the HCP-to-FCC phase transformation. A further orientation analysis indicates that the FCC-Zr phase adopts a B-type orientation relationship with the matrix HCP-Zr. This signifies that the long axes of the FCC-Zr are parallel to the basal planes of the matrix. Consequently, numerous parallel lamellae of the FCC-Zr are formed within the same grain. Abundant stacking faults exist at the boundaries of these lamellae, representing intermediate transformation zones during the phase transformation. Meanwhile, due to the different opening directions of Shockley partial dislocations relative to the nucleation points after nucleation, as well as the varying sizes of the formed FCC-Zr, two variants have emerged: isolated distribution and adjacent distribution. The adjacent variants exhibit a twinning relationship, as illustrated in Figure 9b.

## 4. Conclusions

This study investigates the fatigue damage on the surface of industrial pure Zr specimens under high-cycle fatigue conditions at room temperature with a stress ratio R of 0.1. The experiment focuses on the microscopic characterization of fatigue-induced HCP-to-FCC phase transformation and extensively discusses the formation mechanisms of FCC-Zr lamellae in different variants. The inferences that can be made are as follows:

(1) Through TEM and on-axis TKD characterization, fatigue damage occurs in the form of HCP-to-FCC phase transformation, resulting in the FCC-Zr lamellae. The fatigue-induced FCC-Zr lamellae correspond to the B-type transformation relationship with the HCP-Zr matrix.
(2) For the B-type HCP-to-FCC phase transformation, the formation of two variants results from the different growth directions of the Shockley partial dislocations in relation to the nucleation point. The content of the two variants accounts for 65% and 35% of the total FCC-Zr phase, respectively.
(3) The two variants can either exhibit adjacent distribution or isolated distribution. The two variants with adjacent distribution show a twinning relationship between them. At the boundaries of the FCC-Zr lamellae, stacking faults are observed as an intermediate transition stage of the HCP-to-FCC phase transformation process.

## Data Availability

Not applicable.

## References

[1] Edalati K., Horita Z. (2011). High-pressure torsion of pure metals: Influence of atomic bond parameters and stacking fault energy on grain size and correlation with hardness. Acta Mater..

[2] Jie J., Baifeng L., Dongping X., Ying Z., Jun Z., Jianjun Z. (2015). Study on Deformation Structure and Texture of Pure Zirconium with Large Grain Size Rolled at Liquid Nitrogen Temperature. Rare Met. Mater. Eng..

[3] Srinivasarao B., Zhilyaev A.P., Pérez-Prado M.T. (2011). Orientation dependency of the alpha to omega plus beta transformation in commercially pure zirconium by high-pressure torsion. Scr. Mater..

[4] Zhu Y., Kanamori K., Moitra N., Kadono K., Nakanishi K. (2016). Metal zirconium phosphate macroporous monoliths: Versatile synthesis, thermal expansion and mechanical properties. Microporous Mesoporous Mater..

[5] Low T.S., Niezgoda S.R. (2018). Modeling the α/ω thermal stability in shocked Zr: A coupling between dislocation removal and phase transformation. Acta Mater..

[6] Zhou B.X., Yao M.Y., Li Z.K., Wang X.M., Zhou J., Long C.S., Liu Q., Luan B.F. (2012). Optimization of N18 Zirconium Alloy for Fuel Cladding of Water Reactor. J. Mater. Sci. Technol..

[7] Pandey K., Levitas V.I. (2020). In situ quantitative study of plastic strain-induced phase transformations under high pressure: Example for ultra-pure Zr. Acta Mater..

[8] Guo W., Li G., Han F., Zhang Y., Ali M., Ren J., Wang Q., Yuan F., Tong M. (2022). Deformation mechanism and cyclic stress response of Zircaloy-4 alloy cladding tube during low cycle fatigue at room temperature. Int. J. Fatigue.

[9] Qiu R.S., Luan B.F., Chai L.J., Zhang X.Y., Liu Q. (2014). Effects of heating rates and alloying elements (Sn, Cu and Cr) on the α → α + β phase transformation of Zr–Sn–Nb–Fe–(Cu, Cr) alloys. J. Nucl. Mater..

[10] Tao B., Qiu R., Liu Y., Tan X., Liu Q. (2021). FCC phase transformation of Zr alloy during air cooling and aging. J. Nucl. Mater..

[11] Hu X., Zhao H., Ni S., Song M. (2017). Grain refinement and phase transition of commercial pure zirconium processed by cold rolling. Mater. Charact..

[12] Zhao H., Hu X., Song M., Ni S. (2017). Mechanisms for deformation induced hexagonal close-packed structure to face-centered cubic structure transformation in zirconium. Scr. Mater..

[13] Aguayo A., Murrieta G., De Coss R. (2002). Elastic stability and electronic structure of fcc Ti, Zr, and Hf: A first-principles study. Phys. Rev. B.

[14] Zheng X., Gong M., Xiong T., Ge H., Yang L., Zhou Y., Zheng S., Wang J., Ma X. (2019). Deformation induced FCC lamellae and their interaction in commercial pure Ti. Scr. Mater..

[15] Zhao H., Song M., Ni S., Shao S., Wang J., Liao X. (2017). Atomic-scale understanding of stress-induced phase transformation in cold-rolled Hf. Acta Mater..

[16] Zhang Y., Li G., Yuan F., Han F., Ali M., Guo W., Ren J. (2022). Atomic scale observation of FCC twin, FCC → 9R and 9R → 12R’ transformations in cold-rolled Hafnium. Scr. Mater..

[17] Li Y., Ni S., Liu Y., Song M. (2019). Phase transition induced high strength and large ductility of a hot rolled near β Ti-5Al-5Mo-5V-1Cr-1Fe alloy. Scr. Mater..

[18] Hong D.H., Lee T.W., Lim S.H., Kim W.Y., Hwang S.K. (2013). Stress-induced hexagonal close-packed to face-centered cubic phase transformation in commercial-purity titanium under cryogenic plane-strain compression. Scr. Mater..

[19] Zhang Y., Yuan F., Han F., Ali M., Guo W., Ren J., Li G., Liu C., Gu H., Yuan G. (2021). Direct evidence for stress-induced face-centered cubic zirconium. Mater. Sci. Eng. A.

[20] Niu L., Wang S., Chen C., Qian S.F., Liu R., Li H., Liao B., Zhong Z.H., Lu P., Wang M.P. (2017). Mechanical behavior and deformation mechanism of commercial pure titanium foils. Mater. Sci. Eng. A.

[21] Jiang Q., Chen Y., Shuai Q., Liu F., Li L., He C., Zhang H., Wang C., Liu Y., Wang Q. (2023). Fatigue-induced HCP-to-FCC phase transformation assisting crack nucleation of pure zirconium in high cycle fatigue regime. Mater. Sci. Eng. A.

[22] (2017). Metallic Materials—Fatigue Testing—Axial Force-Controlled Method.

[23] Miao J., Pollock T.M., Jones J.W. (2012). Microstructural extremes and the transition from fatigue crack initiation to small crack growth in a polycrystalline nickel-base superalloy. Acta Mater..

[24] Tofique M.W., Bergstroem J., Svensson K., Johansson S., Peng R.L. (2017). ECCI/EBSD and TEM analysis of plastic fatigue damage accumulation responsible for fatigue crack initiation and propagation in VHCF of duplex stainless steels. Int. J. Fatigue.

[25] Wang Q.Y., Bathias C., Kawagoishi N., Chen Q. (2002). Effect of inclusion on subsurface crack initiation and gigacycle fatigue strength. Int. J. Fatigue.

[26] Akhtar A. (1975). Prismatic slip in zirconium single crystals at elevated temperatures. Metall. Trans. A.

[27] Capolungo L., Marshall P.E., McCabe R.J., Beyerlein I.J., Tomé C.N. (2009). Nucleation and growth of twins in Zr: A statistical study. Acta Mater..

[28] Li L., Zhang Z., Shen G. (2015). The influence of grain size on acoustic emission characteristic in commercial-purity zirconium during tensile deformation. Mater. Sci. Eng. A.

[29] Zhao H., Ding N., Ren Y., Xie H., Yang B., Qin G. (2019). Shear-induced hexagonal close-packed to face-centered cubic phase transition in pure titanium processed by equal channel angular drawing. J. Mater. Sci..

[30] Yang F., Yin S.M., Li S.X., Zhang Z.F. (2008). Crack initiation mechanism of extruded AZ31 magnesium alloy in the very high cycle fatigue regime. Mater. Sci. Eng. A.

[31] Liu Y.G., Li M.Q., Liu H.J. (2016). Surface nanocrystallization and gradient structure developed in the bulk TC4 alloy processed by shot peening. J. Alloys Compd..

[32] Ren Y., Han B., Wu H., Wang J., Liu B., Wei B., Jiao Z., Baker I. (2023). Copper segregation-mediated formation of nanotwins and 9R phase in titanium alloys produced by laser powder bed fusion. Scr. Mater..

[33] Yang C., Li M.Q., Liu Y.G. (2022). Characterization of face-centered cubic structure and deformation mechanisms in high energy shot peening process of TC17. J. Mater. Sci. Technol..

[34] Yang J.X., Liu L.C., Gong H.R., Song M. (2018). Proposed mechanism of twin formation during hexagonal-close-packed structure to face-centered-cubic phase transition. Solid State Commun..

[35] Bruinsma R., Zangwill A. (1985). THEORY OF THE HCP-FCC TRANSITION IN METALS. Phys. Rev. Lett..

[36] Tahara M., Kanaya T., Kim H.Y., Inamura T., Hosoda H., Miyazaki S. (2014). Heating-induced martensitic transformation and time-dependent shape memory behavior of Ti–Nb–O alloy. Acta Mater..

[37] Guo W., Zhang Y., Ren J., Ali M., Wang Q., Yuan F., Han F., Li G. (2023). Atomic scale understanding of the mutual transformations of 2H, 4H, 12R and 3C structures in face-centered cubic zirconium. J. Nucl. Mater..

[38] Tolédano P., Krexner G., Prem M., Weber H.P., Dmitriev V. (2001). Theory of the martensitic transformation in cobalt. Phys. Rev. B.

[39] Liu C., Li G., Yuan F., Han F., Ali M., Zhang Y., Guo W., Gu H. (2020). Core-shell structured nanoprecipitates in zirconium based alloy. Scr. Mater..

[40] Sneddon G.C., Trimby P.W., Cairney J.M. (2016). Transmission Kikuchi diffraction in a scanning electron microscope: A review. Mater. Sci. Eng. R Rep..

[41] Fundenberger J.J., Bouzy E., Goran D., Guyon J., Morawiec A., Yuan H. (2015). Transmission Kikuchi Diffraction (TKD)via a horizontally positioned detector. Microsc. Microanal..

[42] Fundenberger J.J., Bouzy E., Goran D., Guyon J., Yuan H., Morawiec A. (2016). Orientation mapping by transmission-SEM with an on-axis detector. Ultramicroscopy.

[43] Niessen F., Burrows A., Fanta A. (2018). A systematic comparison of on-axis and off-axis transmission Kikuchi diffraction. Ultramicroscopy.

[44] Sokurskii I.N., Protsenko L.N. (1958). Deformation systems of ?-zirconium. Sov. J. At. Energy.

[45] Fu Z., Gao B., Li X., Li C., Pan H., Niu H., Zhu Y., Zhou H., Zhu X., Wu H. (2023). Improved strength-ductility combination of pure Zr by multi-scale heterostructured effects via rotary swaging and annealing. Mater. Sci. Eng. A.

[46] Burgers W.G. (1934). On the process of transition of the cubic-body-centered modification into the hexagonal-close-packed modification of zirconium. Physica.

[47] Sun H., Liang Y., Li G., Zhang X., Wang S., Huang C. (2021). Dislocation hardening and phase transformation-induced high ductility in Ti-6Al-4V with a heterogeneous martensitic microstructure under tensile load. J. Alloys Compd..

[48] Wei B., Ni S., Liu Y., Song M. (2019). Three dimensional crystallographic orientation relationships for hexagonal close packed structure to face centered cubic structure transformation in pure titanium. Scr. Mater..

[49] Xu D.K., Han E.H. (2013). Relationship between fatigue crack initiation and activated twins in as-extruded pure magnesium. Scr. Mater..

[50] Yang F., Lv F., Yang X.M., Li S.X., Zhang Z.F., Wang Q.D. (2011). Enhanced very high cycle fatigue performance of extruded Mg–12Gd–3Y–0.5Zr magnesium alloy. Mater. Sci. Eng. A.

[51] Crépin J., Bretheau T., Caldemaison D. (1995). Plastic deformation mechanisms of β treated zirconium. Acta Met. Mater..

[52] Zhang Y., Li G., Liu C., Yuan F., Han F., Ali M., Guo W., Gu H. (2019). The effect of three-dimensional loading and texture on deformation mechanism of Zircaloy-4 alloy: Using space Schmid factor model. Mater. Sci. Eng. A Struct. Mater. Prop. Microstruct. Process..

[53] An X., Zhang H., Ni S., Ou X., Liao X., Song M. (2020). Effects of temperature and alloying content on the phase transformation and {101¯1} twinning in Zr during rolling. J. Mater. Sci. Technol..

[54] Chen C., Qian S., Wang S., Niu L., Liu R., Liao B., Zhong Z., Lu P., Li P., Cao L. (2018). The microstructure and formation mechanism of face-centered cubic Ti in commercial pure Ti foils during tensile deformation at room temperature. Mater. Charact..

[55] Kou W., Sun Q., Xiao L., Sun J. (2019). Plastic deformation-induced HCP-to-FCC phase transformation in submicron-scale pure titanium pillars. J. Mater. Sci..

[56] Zhu W., Kou W., Tan C., Zhang B., Chen W., Sun Q., Xiao L., Sun J. (2020). Face centered cubic substructure and improved tensile property in a novel β titanium alloy Ti–5Al–4Zr–10Mo–3Cr. Mater. Sci. Eng. A.

